# Understanding the Response of Nitrifying Communities to Disturbance in the McMurdo Dry Valleys, Antarctica

**DOI:** 10.3390/microorganisms8030404

**Published:** 2020-03-13

**Authors:** Maria Monteiro, Mafalda S. Baptista, Joana Séneca, Luís Torgo, Charles K. Lee, S. Craig Cary, Catarina Magalhães

**Affiliations:** 1 School of Science, University of Waikato, 3240 Hamilton, New Zealand; mariaroviscomonteiro@gmail.com (M.M.); charles.lee@waikato.ac.nz (C.K.L.); craig.cary@waikato.ac.nz (S.C.C.); 2 International Centre for Terrestrial Antarctic Research, University of Waikato, 3240 Hamilton, New Zealand; mbaptista@ciimar.up.pt; 3 Interdisciplinary Centre of Marine and Environmental Research (CIIMAR/CIMAR), University of Porto, 4450-208 Matosinhos, Portugal; joanaseneca@gmail.com; 4 LIAAD-INESC Porto LA, R. Ceuta 118 - 6., 4050-190 Porto, Portugal; ltorgo@dal.ca; 5 Faculty of Computer Science, Dalhousie University, 6050 University Av., Halifax, NS N3H 1W5, Canada; 6 Faculty of Sciences, University of Porto, 4169-007 Porto, Portugal; 7 Ocean Frontier Institute, Dalhousie University, B3H4R2 Halifax, Nova Scotia, Canada

**Keywords:** McMurdo Dry Valleys, nitrogen cycle, 16S rRNA gene, 16S rRNA transcripts, nitrifying communities, ammonia oxidation, manipulative experiment

## Abstract

Polar ecosystems are generally limited in nitrogen (N) nutrients, and the patchy availability of N is partly determined by biological pathways, such as nitrification, which are carried out by distinctive prokaryotic functional groups. The activity and diversity of microorganisms are generally strongly influenced by environmental conditions. However, we know little of the attributes that control the distribution and activity of specific microbial functional groups, such as nitrifiers, in extreme cold environments and how they may respond to change. To ascertain relationships between soil geochemistry and the ecology of nitrifying microbial communities, we carried out a laboratory-based manipulative experiment to test the selective effect of key geochemical variables on the activity and abundance of ammonia-oxidizing communities in soils from the McMurdo Dry Valleys of Antarctica. We hypothesized that nitrifying communities, adapted to different environmental conditions within the Dry Valleys, will have distinct responses when submitted to similar geochemical disturbances. In order to test this hypothesis, soils from two geographically distant and geochemically divergent locations, Miers and Beacon Valleys, were incubated over 2 months under increased conductivity, ammonia concentration, copper concentration, and organic matter content. Amplicon sequencing of the 16S rRNA gene and transcripts allowed comparison of the response of ammonia-oxidizing Archaea (AOA) and ammonia-oxidizing Bacteria (AOB) to each treatment over time. This approach was combined with measurements of ^15^NH_4_^+^ oxidation rates using ^15^N isotopic additions. Our results showed a higher potential for nitrification in Miers Valley, where environmental conditions are milder relative to Beacon Valley. AOA exhibited better adaptability to geochemical changes compared to AOB, particularly to the increase in copper and conductivity. AOA were also the only nitrifying group found in Beacon Valley soils. This laboratorial manipulative experiment provided new knowledge on how nitrifying groups respond to changes on key geochemical variables of Antarctic desert soils, and we believe these results offer new insights on the dynamics of N cycling in these ecosystems.

## 1. Introduction

The McMurdo Dry Valleys (MDVs) are the largest contiguous and permanently ice-free area on the Antarctic continent [1] and are regarded as an important area for ecological conservation and protection [2]. Over the past million years, the landscape around the transantarctic mountains has been determined by a long history of environmental change that altered the geography, leaving behind massively mixed glacial tills of heterogeneous soils containing numerous ice-covered lakes, ponds, glaciers, and associated meltwater streams [1]. Soils are extremely dry and salty due to low levels of precipitation combined with the evaporation and sublimation of falling snow [1]. The lack of liquid water and the physical isolation of the continent limit the development of complex biological communities, resulting in ecosystems with simple trophic interactions [3,4] and mostly composed of microorganisms. These microbial communities play a fundamental role in nutrient turnover and energy flow through the system [5].

Nitrogen (N) is generally limited in the Dry Valleys [6]. The majority of biological N input in the system is dependent on the activity of N-fixing bacteria such as cyanobacteria and heterotrophic diazotrophs [7,8,9,10], whose distribution and activity are driven by moisture gradients, being mostly abundant in the vicinities of ponds, lakes, and the hyporheic zones [8]. Nitrogen can also be sourced from the atmospheric deposition of nitrate salts, which tend to accumulate in dry and high-altitude valleys due to the absence of vegetation as well as to the aerobic conditions of the soils which inhibit reductive pathways of the N cycle, such as denitrification [11]. As such, nitrification may be an important biological process for N cycling and the productivity of terrestrial polar systems [12,13,14]. Nitrification comprises two main reactions, generally carried out by different microorganisms. The first step, the oxidation of ammonia into nitrite (NO_2_^−^), is carried out by ammonia-oxidizing archaea (AOA) and bacteria (AOB) [15,16]. The second step, the oxidation of NO_2_^-^ into nitrate (NO_3_^−^), is carried out by nitrite-oxidizing bacteria (NOB) [17]. It is now known that some NOB possess the enzymatic repertoire necessary to catalyze both reactions and can perform the full nitrification process [18]. 

Various environmental factors have been related to the distribution and activity of AOA and AOB in different ecosystems, yet these analogous functional groups can be composed of different and dynamic populations or ecotypes adapted to microenvironments within the system [19,20,21]. Therefore, the diversity of the community can potentially help to neutralize the effects of disturbance through spatial or temporal rearrangements of these smaller populations, with important implications for the process stability [22]. Although not extensively analyzed, the presence/absence, diversity, and structure of nitrifying communities have been assessed in different regions of Antarctica, namely in continental shelf waters and across a latitude gradient in the Antarctic Peninsula [7,12] and in the McMurdo Dry Valleys soils and lakes from the Ross Sea region [14,23,24]. These studies have concluded that environmental variables such as water availability and soil geochemistry are correlated with the distribution, abundance, and diversity of different nitrifying groups and could be driving a niche specialization and differentiation between AOA and AOB communities in the soils [14]. However, field surveys are unable to discern the response of analogous nitrifying groups to the alteration of those variables and their implications to the nitrification process [25]. 

To gain a deeper insight on the relationship between soil geochemistry, AOA and AOB activity, and relative abundance, we set up a controlled manipulation experiment using soils from two distant valleys within the MDVs: Miers and Beacon Valleys. The choice of these two valleys was based on their contrasting soil geochemistry [26] and abundance of nitrifying groups [14]. Miers Valley is a coastal valley with higher C/N ratio and lower electrical conductivity (EC) relative to Beacon Valley. Beacon Valley is located at a much higher altitude (1376 vs 171 m for Miers Valley) and subject to lower temperatures and stronger winds. Long-term atmospheric salt deposition (e.g., NO_3_^−^), weathering events, and the absence of precipitation are likely responsible for the much higher electrical conductivity in Beacon Valley soils [11,27] and make Beacon Valley a much more challenging microbial habitat compared to Miers Valley [14,26,28]. 

In this study, we tested the hypothesis that nitrifying communities, adapted to different environmental conditions within the MDVs, have distinct responses when subjected to similar geochemical disturbances. To address our hypothesis we designed a manipulative experiment to assess the response of AOA and AOB from two McMurdo Dry Valleys, to the alteration of conductivity, ammonia concentration, Cu concentration, and organic matter content. The potential impact on the nitrification pathway in both valleys was monitored along the incubation time using the relative abundances of nitrifying taxa through 16S rRNA transcripts and 16S rRNA gene sequencing and ^15^NH_4_^+^ oxidation rates as a proxy for nitrification. Conductivity, Cu, and organic matter were chosen based on previous study results [14,26]. In situ studies have identified higher AOA/AOB ratios when conductivity and Cu contents in the soils were also higher [14]. Moreover, higher conductivity and Cu contents in Beacon Valley and higher content of carbon (C) in Miers Valley were significantly correlated with differences in microbial community structure and functional capacity [26,29,30]. 

## 2. Materials and Methods

### 2.1. Site Description 

Soils were collected during a 2013 expedition to the MDVs under the New Zealand Terrestrial Antarctic Biocomplexity Survey (nzTABs) project (available online: https://ictar.aq/nztabs-science/). A bulk soil sample was collected from 1 to 5 cm depth in Miers Valley and Beacon Valley, sealed, and kept at −20 °C until further analyses, as described in [26]. Miers Valley (78°05.486′S, 163°48.539′E, elevation 171 m) is a coastal valley located at the southern end of the MDVs [28,31]. It is a granite-dominated valley, without any vegetation and with floors characterized by moraine deposition, steep scree, and boulder slopes [32]. Compared to Beacon Valley, Miers valley has less severe environmental weathering, and the soils have lower conductivities and generally higher C/N ratios [26]. Beacon Valley (77°52.321′S, 160°29.725′E, elevation 1376 m) is one of the highest ice-free valleys in the MDVs, and it is located close to the polar plateau [28,31]. Its geomorphology shows clear evidence of several past glacial events with a flat valley floor covered with tills of dolerite, sandstone, granitic boulders, cobbles, and pebbles [33]. Vegetation is absent, and the soils are ultra-oligotrophic, with high conductivities due to long-term atmospheric salt deposition and low precipitation [26].

### 2.2. Manipulative Experimental Setup 

In September 2015, the soils from Miers and Beacon Valleys were thawed, sieved (2 mm), and homogenized. Subsequently, 40 g of sieved and homogenized soil was placed in 50 mL falcon tubes. Four different treatments were performed in triplicate, consisting of adding a 13 mL solution of Treatment 1, 15 µM NH_4_Cl, termed NH_4_; Treatment 2, 200 µM CuCl_2_, termed Cu; Treatment 3, addition of a NaCl solution with an appropriate concentration to mimic an increase of electric conductivity of 3000 µS/cm, termed NaCl; and Treatment 4, 300 µM C_6_H_12_O_6_, to mimic an increase of organic compounds in the soils [29], termed glucose. For all treatments a ratio of ^15^NH_4_Cl and ^14^NH_4_Cl (3:2 ratio) was added to a final concentration of 4 µM NH_4_^+^, and 15 µM NH_4_^+^ (Treatment 1), in order to measure NH_4_^+^ oxidation rates (see detailed description below). Thus, the control consisted in treating the sample solely with 4 µM NH_4_^+^ at similar ^15^N/^14^N ratio. MiliQ water was used to make the solutions, and all chemicals used were of Pro Analysis grade or equivalent.

Soils were incubated at 4 °C in the dark in falcon tubes, which were gently mixed to maintain homogeneous aerobic conditions across the soil. Samples were collected after 1, 28, and 65 (Beacon) or 68 (Miers) days of incubation. At the time of sampling, one falcon tube per treatment was destructively sampled. RNA extraction was performed on the same day as the sampling, and the remaining soil was divided according to the different analysis described below and stored at −20 °C until used. 

### 2.3. Inorganic Nitrogen Analysis 

From each falcon tube 20 g of soil was extracted with 50 mL of 2 M KCl. Samples were stirred for 1 h at 180 rpm [34]. The overlaying solution was subsequently filtered through 0.45 µm cellulose acetate filters and stored at −20 °C until analysis. The concentration of inorganic N species (^15^NH_4_^+^, NO_2_^−^ + NO_3_^−^) was determined spectrophotometrically and in duplicate for each sample, according to previously described methods [35].

### 2.4. ^15^NH_4_^+^ Oxidation Rate Measurements 

Oxidation rates of ^15^NH_4_*^+^* were measured by the addition of 2.4 µM of ^15^NH_4_Cl (>95% ^15^N Sigma-Aldrich, Madrid, Spain) plus 1.6 µM of NH_4_Cl, to a final concentration of 4 µM, in every treatment. The ^14/15^NO_2_^−^ + **^14/15^**NO_3_^−^ pool was extracted in duplicate as described before for the inorganic nutrients. Filtered samples were reduced using an adaptation of the spongy cadmium method [36] and centrifuged at 1520x *g* for 10 min. Four milliliters were transferred to a 10 mL exetainer and purged with Argon (≥ 99.9%) for 10 min to remove any background N_2_ [37]. Fifty microliters of sulfamic acid (4%) of each replicate was injected in each exetainer [38], followed by shaking and overnight incubation, with the exetainer inverted, in the dark at room temperature. Analysis of the N_2_ produced was performed by EA-IRMS as described in Santos et al. [39]. The potential for nitrification was considered as the ^15^NH_4_*^+^* oxidation rates (pmol × g^−1^ × d^−1^) calculated from the slopes of the linear regressions of ^15^N concentration vs time [40].

### 2.5. DNA Extration

Total DNA was extracted at each time point, from two replicates of 0.8 g of soil using bead-beating method [4,26] and CTAB (bromide-polyvinylpyrrolidone-b-mercaptoethanol) as described previously [14]. The extraction duplicates were pooled and the concentration determined using the Qubit dsDNA HS Assay Kit (ThermoFisher Scientific, Auckland, New Zealand).

### 2.6. RNA Extration and Reverse Transcription

To increase the chances of a successful RNA extraction from soils with a low biomass content, total RNA was isolated from 15 g of homogenized soil using RNA PowerSoil kit from MO BIO (Carlsbad, CA, USA) with proportional adjustments on the reaction volumes of the kit. The concentration of total RNA was determined using the Qubit RNA HS Assay Kit (ThermoFisher Scientific, Auckland, New Zealand). RNA products were immediately treated with DNase (TURBO™ DNase, Ambion Life Technologies) to remove any residual DNA and subsequently reverse-transcribed into cDNA using Thermoscript RT_PCR kit with random primers (ThermoFisher Scientific, Auckland, New Zealand), according to manufacturer’s protocol. The persistence of genomic DNA contamination in total RNA was checked by PCR using total RNA sample as a template and the primer set 806R/515F [41].

### 2.7. Amplicon Sequencing

Preparation for sequencing started with amplification of the V4 region of the 16S rRNA gene, using 1 ng of DNA or cDNA and the 806R/515F-barcoded primer set pair [41]. The PCR master mix was treated with ethidium monoazide (EMA) prior to amplification to prevent exogenous DNA contamination (Nocker and Camper, 2006) [42]. PCR reactions were performed in triplicate (25 mL reaction volume) and included 10X PCR buffer, 0.2 mM dNTPs, 0.4 mg/mL bovine serum albumin (BSA), 3 mM MgCl2, 1 unit of Platinum Taq (Invitrogen Inc., 75 Carlsbad, CA, USA), 10 mM of each primer, and UV RNA/DNA-free water. The thermocycler program used was as follows: 94 °C for 3 min, followed by 30 cycles of 94 °C for 45 sec, 50 °C for 1 min, 72 °C for 1.5 min, and a final extension at 72 °C for 10 min. PCR products were pooled and visualized on a 1% agarose gel. After amplification, PCR products were cleaned with SPRI select (Beckman Coulter, Inc, Auckland, New Zealand) to remove primer dimers. The SPRI-cleaned PCR products were then quantified with Qubit dsDNA HS assay (ThermoFisher Scientific, Auckland, New Zealand). To make the final equimolar library, each sample was diluted to 26 pM, and an equal volume of each sample was then pooled. Sequencing was performed with an Ion PGM™ System at the Waikato DNA Sequencing Facility (University of Waikato, Hamilton, New Zealand). Ion PGM™ Template IA 500 Kit was used for template preparation with the Ion OneTouch™ Enrichment System, and Ion PGM™ Hi-Q™ Sequencing Kit was used with the Ion 318™ Chip v2, following the manufacturer’s protocols.

### 2.8. Sequencing Analyses

Raw sequences were filtered with PGM software to remove low-quality and polyclonal reads. The remaining sequences were processed using a combination of Mothur and Usearch pipelines [43,44,45]. Primers were identified within the sequence and trimmed using the FASTX toolkit. Sequences in which the primers were not identified were discarded. The remaining sequences were trimmed based on length and the number of homopolymers sourced by Mothur script [43]. Sequences were truncated at 250 bp and submitted for quality filtering using the Q scores of fastQ files. Through dereplication, unique sequences were identified, and their abundances quantified. Sequences were sorted and the singletons removed. The remaining sequences were clustered into representative OTUs using UPARSE and uchime algorithms, in combination with the GOLD database to detect and remove chimeras [44,45,46]. Reads were then clustered into operational taxonomic units (OTUs) using a similarity threshold of 97%. Sequences that did not map to any OTU were discarded. Taxonomy was inferred using the RDP classifier at 80% identity threshold [47]. The identification of nitrifying organisms was performed using RDP taxonomy assignments at a confidence level higher than 80%.

Sequencing efforts (16S rRNA gene and 16S rRNA transcripts) resulted in an average of 19,130 sequences per sample after quality filtering. Raw sequences were deposited in NCBI under the SRA accession PRJNA527658.

### 2.9. Statistical Analysis

A Dunnett’s test was conducted to test for pairwise differences in inorganic N concentrations between each treatment and the control, and accumulation of inorganic N was calculated from the slopes of the linear regressions of concentration vs time. Non-metric multidimensional scaling ordination allowed the separation of the data by time points. After testing for the multivariate homogeneity of group dispersions, a PERMANOVA was used to inspect significant differences between those groups. Statistical analyses were computed with R [48] using “stats” and “vegan” packages [49]. Graph plotting was computed using “phyloseq” and “ggplot2” libraries [50,51].

## 3. Results and Discussion

### 3.1. Resident and Active Microbial Communities from Miers and Beacon Valleys

**Figure 1:**
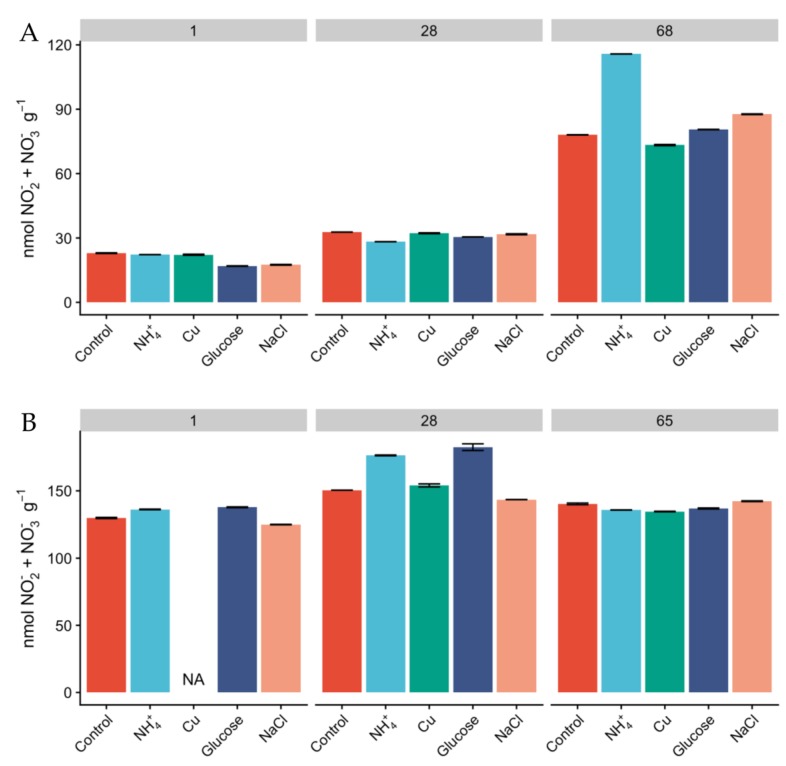
Concentration of NO*_2_*^−^ + NO*_3_*^−^ (nmol NO*_2_*^−^ + NO*_3_*^−^ g^−1^) in Miers (**A**) and Beacon (**B**) Valley soils over the course of the experiment, along the different treatments. Columns represent mean values, and bars are the standard deviations of two replicates.

Milder conditions observed in Miers Valley are reflected in the presence of a higher microbial biomass and diversity [14,26,28]. In our study, total DNA yields for Miers Valley were consistently higher than for Beacon Valley, averaging 415 ± 310 and 192 ± 141 ng per gram of soil respectively (Table S1), in agreement with previous observations [14]. Total RNA yields ranged between 37 to 159 ng per gram of soil for Miers Valley and between 4 to 27 ng per gram of soil for Beacon Valley. Notably, RNA extraction was unsuccessful for most of the Beacon Valley soil samples (Table S1). The use of rRNA gene as a proxy for microbial activity is subject to some limitations [52]. Nevertheless, a three-fold difference in total RNA yields between soils from Miers Valley and Beacon Valley can be an indication that microbial communities are potentially more active in Miers Valley soils. Successful RNA extractions were only achieved for Beacon Valley soils after 28 days of incubation, potentially indicating that soil microbial communities from Beacon Valley naturally show very low levels of activity. These results corroborate an earlier study of the adjacent University Valley, which reported extremely low microbial biomass and complete absence of microbial activity [53]. However, our results also suggest that the extant microbial communities in Beacon Valley soils are viable and capable of responding to change. Along with the lower DNA and RNA yields in Beacon Valley, the lack of variation of NO_2_^−^ + NO_3_^−^ in Beacon soils (Figure 1) contrasted to its linear increase in Miers Valley soils and suggested a low turnover of N-species, which could also indicate that the microbial communities involved in N cycling might not have been very active during the manipulation in Beacon Valley.

### 3.2. The Distinct Potential for Nitrification Between Miers and Beacon Soils

The potential for nitrification from the soil microbial communities in Miers and Beacon Valleys was assessed by linking results from measurements of inorganic N concentrations and ^15^NH_4_^+^ oxidation rates obtained throughout the incubation period, with the taxonomy and relative abundance of nitrifying taxa through 16S rRNA gene sequencing and quantification at the DNA and cDNA levels. The use of 16S rRNA transcript cDNA amplicons offers an extra layer of information by inferring the abundance of active microorganisms within a community [54]. However, due to the unsuccessful extraction of RNA in most Beacon Valley samples, we used 16S rRNA gene PCR amplicon sequences for this valley in downstream analyses.

Our attempts to quantify the *amo*A gene by qPCR in the cDNA were not successful for most of the samples, possibly due to the low expression levels. Therefore, it was not possible to draw definitive conclusions regarding the activity of ammonia-oxidizing groups in the manipulative experiments we set up in Beacon Valley samples, apart from the results from the potential nitrification rates. Yet, it was possible to describe trends and compare the response of bacterial and archaeal ammonia-oxidizing groups to the same treatments in the two different soils.

Analysis of 16S rRNA gene PCR amplicons in Miers and Beacon Valley showed that nitrifying organisms comprised 0.35% of the sequences in Miers Valley soils before the start of the experiment, whereas no sequences affiliated with known nitrifying groups could be identified in Beacon Valley (Supplementary Material Figure S1A). Analysis of 16S rRNA transcript cDNA amplicons from Miers Valley showed that nitrifying organisms comprised 0.14% of the sequences associated with the active community before the start of the experiment (Supplementary Material Figure S1A).

**Figure 2:**
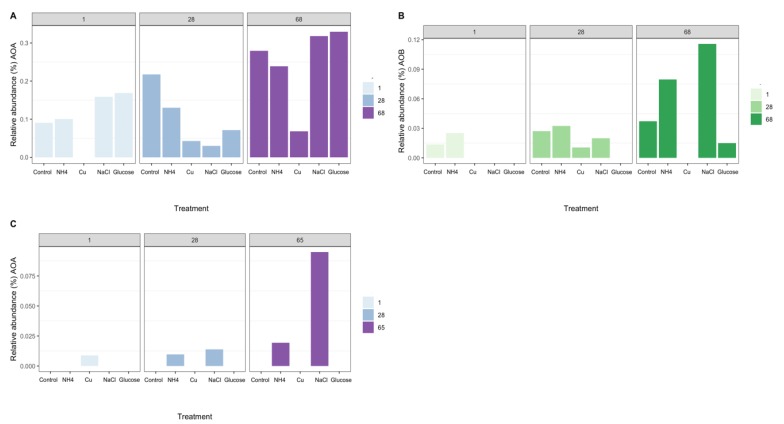
Relative abundance (%) of 16S rRNA transcript cDNA amplicons (Miers Valley—**A**,**B**) and 16S rRNA gene PCR amplicons (Beacon Valley—**C**) assigned to known nitrifying organisms over the course of the experiment and across the different treatments. (**A**)—Relative abundance (%) of archaeal 16S rRNA transcripts, assigned to ammonia-oxidizing archaea (AOA), in Miers Valley soils over the course of the experiment, between the different treatments. (**B**)—Relative abundance (%) of 16S rRNA transcripts, assigned to ammonia-oxidizing bacteria (AOB), in Miers Valley soils over the course of the experiment, between the different treatments. (**C**)—Relative abundance (%) of archaeal 16S rRNA gene, assigned to ammonia-oxidizing archaea (AOA), in Beacon Valley soils over the course of the experiment across the different treatments. The lack of bars represents treatments for which no reads were retrieved.

The nitrifying community in Miers Valley soils was composed of two OTUs affiliated with the genus *Nitrososphaera* [55], two OTUs affiliated with the family *Nitrosomonadaceae* [56], and two OTUs affiliated with genus *Nitrospira* [57] (Figure 2A,B). In Beacon Valley soils only one OTU affiliated with the genus *Nitrososphaera* was detected in day one for the Cu treatment and after 28 days on the conductivity and ammonia treatments. The maximum relative abundance of this OTU was observed after 65 days of incubation in the NaCl treatment (Figure 2C). No AOB nor NOB were identified in Beacon Valley soils during the manipulation experiment. Even though we failed to detect AOB and NOB, previous direct surveys successfully identified and quantified AOB in Beacon valley soils [14] by specifically targeting the bacterial *amoA* gene. Yet, the abundance of bacterial *amo*A gene was significantly lower than its archaeal counterpart in Beacon Valley soils, and the same was true for other more environmentally extreme valleys, such as the Upper Wright Valley [14]. In other ecosystems, AOA have been reported as the more prominent nitrifying group (relative to AOB) under more extreme environmental conditions, such as oligotrophy, low ammonium concentration, [14,21], and low levels of dissolved oxygen [14,19,58], indicating a higher adaptability of this functional group to harsher environmental pressures.

**Figure 3:**
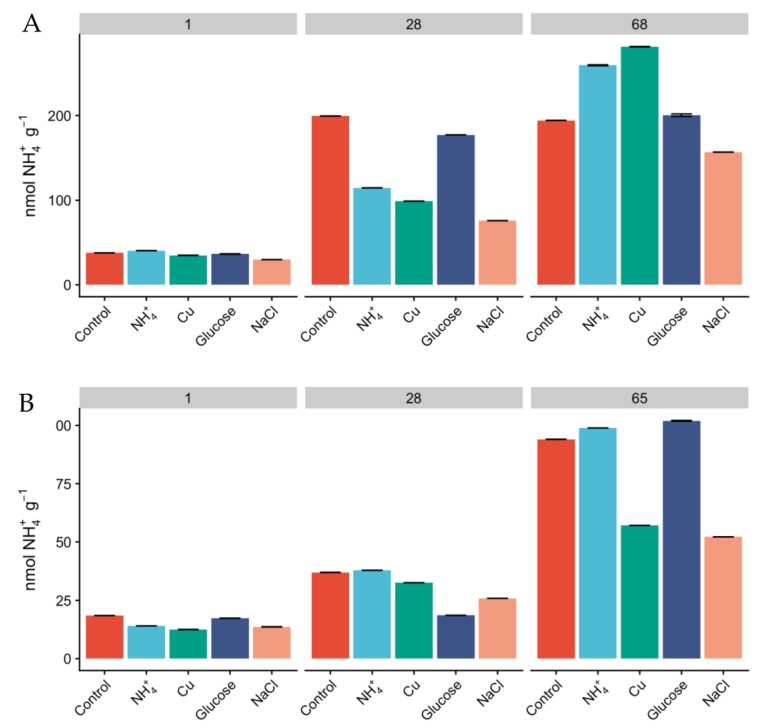
Concentration of ^15^NH_4_^+^ (nmol **^1^**^5^NH_4_^+^ g^−1^) in Miers (**A**) and Beacon (**B**) Valley soils over the course of the experiment, across the different treatments. Columns are mean values, and bars are the standard deviations of two replicates.

Since the relative abundances of AOA and AOB do not necessarily reflect activity [25], we measured oxidation rates of ^15^NH_4_^+^ as a proxy for nitrification activity and monitored the concentration of inorganic N pools over the course of the experiment. The concentration of NH4^+^ was higher in Miers Valley soils than that in Beacon Valley after 24 h, and NH_4_^+^ showed a linear increase over time across all treatments (Figure 3). This could potentially be a result of increasing mineralization rates and cellular degradation through microbial activity [59]. Since the rates of microbial heterotrophic activity and nitrification are expected to be low in the Dry Valleys [60], the accumulation of NH_4_^+^ in the soils was expected to be low (Figure 3). None of the treatments displayed significant differences in the accumulation of NH_4_^+^ over time compared to the control, but after two months of incubation the concentration of NH_4_^+^ was higher than at day 1 for every treatment. The concentration of NO_2_^−^ + NO_3_^−^ was naturally higher in Beacon Valley compared to that of Miers Valley (Table 1). This could be a reflection of atmospheric deposition of salts in this high-altitude valley [11,27]. However, it showed no significant variation over time nor between treatments (Figure 1). Conversely, there was a steep, linear increase of NO_2_^−^ + NO_3_^−^ in Miers Valley soils (Figure 1). The lack of variation in NO_2_^−^ + NO_3_^−^ in Beacon soils suggests a low turnover of N-species, which could indicate that the microbial community involved with the production/consumption of NO_2_^−^ + NO_3_^−^ might not have been very active during the experiment manipulation, corroborating the low RNA yields and the low relative abundance of nitrifying taxa.

**Figure 4:**
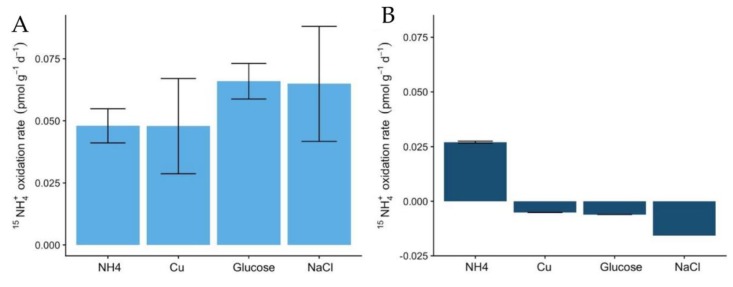
Potential ammonia oxidation rates (pmol g^−1^ d^−1^) using labelled *^15^*NH*_4_*^+^ to a final concentration of 4 µM in Miers (**A**) and Beacon Valleys soils (**B**) for the following treatments: ammonium augmentation (NH*_4_*^+^), Cu and glucose amendments, and conductivity treatment. Error bars indicate the standard variation in the rates measured of two replicates.

The rates of ^15^NH_4_^+^ oxidation in the soils also indicate the higher potential of Miers Valley soils to perform the nitrification pathway (Figure 4A), compared to Beacon Valley (Figure 4B). These results, associated with the lack of variation of NO_2_^−^ + NO_3_^−^ in Beacon Valley soils, indicate a low potential for nitrification or even the absence of this process. The oxidation rates in Miers Valley soils were low (0.05 pmol × g^−1^ × d^−1^), which is expected for oligotrophic systems [40] and aligns with the low abundance of nitrifying groups in the soils.

### 3.3. Treatment Effects on Nitrifying Communities from Miers and Beacon Soils

The overall effect of the different treatments on the variability of ammonia-oxidizing communities was not significant (PERMANOVA, R^2^ = 0.28, *p* = 0.7); however, there was a significant effect of time on the variability of these communities in Miers Valley (PERMANOVA, R^2^ = 0.64, *p* = 0.03). A possible explanation for this might have been due to the necessary addition of water to amend the soils for each individual treatment. The increase of water availability in Antarctic soils has been suggested to promote significant shifts in the diversity and functionality of the terrestrial microbiome [30], such as the alteration of community composition and increase of respiration rates and primary production [61,62]. Nevertheless, during the course of the experiment we were able to observe distinct responses (in terms of relative abundance) between archaeal and bacterial ammonia-oxidizing taxa to particular treatments (Figure 2).

The initial amendment with NH_4_^+^ did not have a significant effect on the relative abundance of AOA and AOB (Figure 2A,B); however, the observed accumulation of NH_4_^+^ over the course of the manipulation (Figure 3 and Tables S2 and S3) could have triggered an increase on the relative abundance of 16S rRNA transcripts affiliated with ammonia-oxidizing groups in Miers Valley (Figure 2A,B), particularly for AOB since for AOA this increase was not different from the one seen in the control (Figure 2A, 68 days). Nonetheless, compared to other environments, the concentration of NH_4_^+^ was relatively low (Table 1), hence potentially giving an advantage to AOA over AOB, since archaeal ammonia monooxygenase enzymes (AMO) have higher substrate affinities than bacterial AMO [63]. In Beacon Valley, the relative abundance of AOA-affiliated 16S rRNA gene sequences showed a slight increase after 28 and 65 days of incubation (Figure 2C), which could be suggestive of an initial slow adaptation to the environment by this functional group, as seen in other environments [64]. AOB remained undetected over the time in Beacon Valley soils (not represented in Figure 2).

The addition of Cu affected ammonia-oxidizing communities, since neither AOA nor AOB were detected after 1 day of incubation in Miers Valley soils (Figure 2A,B) and in days 28 and 65 in Beacon soils (Figure 2C). The toxic effect of Cu for microbial communities has been previously shown in the Dry Valleys [28].

The addition of glucose as an organic carbon source was the treatment with the most contrasting effect on the two functional ammonia-oxidizing groups. In Miers Valley this treatment triggered an increase in 16S rRNA transcripts affiliated with AOA, whereas it inhibited AOB (Figure 2A,B). Interestingly, this result was followed by a slight increase of ^15^NH_4_^+^ oxidation rates (from 0.05 to 0.07 pmol N g^−1^ d^−1^), which, despite not being significant compared to the control, was the highest rate measured during the experiment (Figure 4). Although AOA are generally considered autotrophs, studies in other ecosystems have reported their ability to use organic carbon sources and carry out mixotrophic metabolism [65,66,67,68]. It is also likely that the addition of a carbon source caused an acceleration of ammonia mineralization, or a positive priming effect, through the stimulation of microbial activity. Such effect was supported by the significant increase in NO_2_^-^ + NO_3_^-^ (Figure 1) in soils subjected to this treatment. Although we do not have enough data to better interpret the influence of organic matter in AOA functionality, the response observed in this study could have significant implications for predicting impacts of climate change on the nitrification process in Antarctic terrestrial systems. In fact, climate change impact is likely to result in an increase of water availability, primary production rates, and ultimately in an increase of organic carbon availability [30].

The only treatment that caused a similar response in both soils was the change in electrical conductivity (EC), which promoted an increase in the relative abundance of ammonia-oxidizing organisms (Figure 2). The effect of EC increase was surprising for Beacon Valley soils, for which a compelling increase of 16S rRNA gene sequences affiliated with AOA was observed after 65 days (Figure 2C). In situ, EC values for Beacon Valley soils are usually ten times higher than that of Miers Valley (Table 1), suggesting that the positive response of AOA in Beacon Valley (days 28 and 65) could be related to a better inherent adaptation of this functional group to high salt concentrations. These results corroborate previous in situ observations where soil conductivity was identified as a factor affecting AOA abundance in Beacon Valley soils [14]. For the EC treatment, the relative abundance of Miers Valley 16S rRNA transcripts affiliated with AOB was three times higher after 68 days relative to the control for the same treatment (Figure 2B) and showed a linear increase throughout the manipulation period. Interestingly, ^15^NH_4_^+^ oxidation rates increased in Miers Valleys soils subjected to an increase in EC (Figure 4), yet this increase was not significant compared to the control.

The composition of ammonia-oxidizing communities has been previously surveyed in the McMurdo Dry Valleys soils and lakes [14,23,24]. These studies demonstrated that the distribution of AOA and AOB in these environments is driven by local edaphic variables. In the present study we complement the previous results by providing the first experimental evidence of an active response of Antarctic ammonia-oxidizing communities to singular geochemical changes. Using this framework we were also able to depict differential responses from two functionally redundant groups to specific changes in Antarctic soils. While this approach might not have captured the entire diversity, nor the absolute abundance of ammonia-oxidizing groups since it did not target and specifically quantify AOA and AOB groups, and the coverage of 16S rRNA gene primers is still limited and biased by the completeness of the database [59], it was still useful to indicate that compositional tradeoffs might occur in the Antarctic soils to maintain geochemical pathways. These could be related to the level of tolerance of certain functional groups to environmental stressors.

## 4. Conclusions

This study provides experimental evidence for the nitrification potential in the soil of two geochemically different Antarctic Dry Valleys as well as the response of archaeal and bacterial ammonia-oxidizing groups to the effects of disparate geochemical variables. Our results show that soils from the Miers Valley, where the environmental conditions are milder, harbor ammonia-oxidizing microorganisms potentially more active and more abundant than those found in the older and more environmentally extreme Beacon Valley. These results were congruent with our geochemical measurements, which showed a higher N turnover as well as measurable rates of ammonia oxidation in Miers Valley soils, neither of which could be detected for Beacon Valley soils. The alteration of conductivity, ammonia concentration, Cu concentration, and glucose content was not reflected on the activity of ammonia-oxidizing groups. Nonetheless, our analysis indicated AOA as the most abundant ammonia-oxidizing functional group in both soils, and the most adaptive to the initial increase in Cu concentration and glucose in Miers Valley soils. AOA were also the only nitrifying group identified in Beacon Valley soils, supporting previous findings that AOA are ubiquitous and thrive under harsh environmental pressures, like high salt concentrations, in terrestrial polar systems.

## Figures and Tables

**Table 1:** Physicochemical properties of Miers and Beacon Valley soils.

	Miers Valley	Beacon Valley	Study
pH	8.62 ± 0.31	7.10 ± 0.28	[26]
Conductivity (µS/cm)	300	3920	[26]
C/N	18.22 ± 20.07	1.80 ± 0.46	[26]
Cu (ppm)	11.9 ± 2.7	73.7 ± 12. 6	[26]
AOA *amoA* gene copies (g^−1^ soil)	6 × 10^5^	1 × 10^5^	[14]
AOB *amoA* gene copies (g^−1^ soil)	1 × 10^6^	5 × 10^3^	[14]
NH_4_^+^ (nmol g^−1^)	22.9	23.5	This study
NO_2_^−^ + NO_3_^−^ (nmol g^−1^)	4.11	28.9	This study

Data from this study are the average of two analytical replicates with relative standard deviation (RSD) below 7%.

## Supplementary Materials

Supplementary materials can be found at https://www.mdpi.com/2076-2607/8/3/404/s1.
