# Supplementary material for: Understanding the Response of Nitrifying Communities to Disturbance in the McMurdo Dry Valleys, Antarctica
Source: Microorganisms. 2020 Mar 13;8(3):404. doi: 10.3390/microorganisms8030404 (PMC7143839; doi:10.3390/microorganisms8030404)
Supplement: Supplementary file 1 [file microorganisms-08-00404-s001.pdf]

Supplementary Information

Understanding the response of nitrifying communities to disturbance in the McMurdo Dry Valleys, Antarctica

Maria Monteiro[1,2], Mafalda S. Baptista[2,3,6], Joana Séneca[3], Luís Torgo[4,5,6], Charles K. Lee[1,2], S. Craig Cary[1,2], Catarina Magalhães[1,3,6,7,*]

[1] School of Science, University of Waikato, Hamilton, New Zealand
[2] International Centre for Terrestrial Antarctic Research, University of Waikato, Hamilton, New Zealand
[3] Interdisciplinary Centre of Marine and Environmental Research (CIIMAR/CIMAR), University of Porto, Portugal
[4] LIAAD-INESC Porto LA, R. Ceuta 118 - 6., 4050-190 Porto, Portugal
[5] Faculty of Computer Science, Dalhousie University, 6050 University Av., Halifax NS, N3H 1W5, Canada
[6] Faculty of Sciences, University of Porto, Porto, Portugal
[7] Ocean Frontier Institute, Dalhousie University, Canada

* Correspondence: cmagalhaes@ciimar.up.pt (CM)

Received: 17 February 2020; Accepted: 10 March 2020; Published: 13 March 2020

Table S1 - Total amount of DNA and RNA per gram of soil in Miers and Beacon Valley soils for each treatment and for each time point.

Sample ID	Valley	Treatment	Days	DNA (ng/g soil)	Total RNA (ng/g soil)
5M	Miers	Control	1	241	52
6M	Miers	Control	28	273	37
7M	Miers	Control	68	*Below detection limit (<0.5 ng/mL)*	39
9M	Miers	Cu	1	418	*Below detection limit (<20 ng/mL)*
10M	Miers	Cu	28	196	119
11M	Miers	Cu	68	355	106
13M	Miers	NaCl	1	850	99
14M	Miers	NaCl	28	712	53
15M	Miers	NaCl	68	154	69
17M	Miers	Glucose	1	822	44
18M	Miers	Glucose	28	458	77
20M	Miers	Glucose	68	307	143
21M	Miers	NH4	1	185	124
22M	Miers	NH4	28	193	171
23M	Miers	NH4	68	333	159
5B	Beacon	Control	1	185	*Below detection limit (<20 ng/mL)*
6B	Beacon	Control	28	163	*Below detection limit*

					(<20 ng/mL)
7B	Beacon	Control	65	81	13
9B	Beacon	Cu	1	186	*Below detection limit (<20 ng/mL)*
10B	Beacon	Cu	28	254	*Below detection limit (<20 ng/mL)*
11B	Beacon	Cu	65	165	4
13B	Beacon	NaCl	1	114	*Below detection limit (<20 ng/mL)*
14B	Beacon	NaCl	28	335	*Below detection limit (<20 ng/mL)*
15B	Beacon	NaCl	65	54	*Below detection limit (<20 ng/mL)*
17B	Beacon	Glucose	1	154	*Below detection limit (<20 ng/mL)*
18B	Beacon	Glucose	28	238	*Below detection limit (<20 ng/mL)*
19B	Beacon	Glucose	65	50	*Below detection limit (<20 ng/mL)*
21B	Beacon	NH4	1	651	*Below detection limit (<20 ng/mL)*
22B	Beacon	NH4	28	173	*Below detection limit (<20 ng/mL)*
23B	Beacon	NH4	65	91	27

Table S2 – Concentration (μM) of inorganic N compounds in Miers valley soils during the manipulative experiment.

**Miers Treatment**	**Time (days)**	**NH_4^+ (μM)**	**sd**	**NO_3^- (μM)**	**sd**	**NO_2^- (μM)**	**sd**
Control	1	15.12	0.58	5.28	0.30	3.89	0.03
Control	28	79.74	0.53	8.81	0.06	4.28	0.00
Control	68	77.66	0.97	27.22	0.12	4.01	0.00
Cu	1	13.96	1.55	4.87	0.42	4.01	0.03
Cu	28	39.62	0.97	9.39	0.36	3.49	0.02
Cu	68	112.55	2.13	25.38	0.48	3.94	0.08
NaCl	1	11.94	0.53	3.81	0.29	3.19	0.21
NaCl	28	30.38	0.48	8.58	0.39	4.11	0.34
NaCl	68	62.67	0.77	30.80	0.29	4.27	0.10
Glucose	1	14.61	2.27	2.45	0.18	4.32	0.05
Glucose	28	70.81	0.29	9.14	0.03	3.05	0.06
Glucose	68	80.15	13.69	28.16	0.15	4.05	0.03
NH4	1	16.18	0.34	4.82	0.03	4.08	0.00
NH4	28	45.90	0.04	8.68	0.03	2.61	0.03
NH4	68	103.76	5.66	42.29	0.03	4.01	0.00

Table S3– Concentration (µM) of inorganic N compounds in Beacon valley soils during the manipulative experiment.

Beacon Treatment	Time (days)	NH_4^+ (µM)	sd	NO_3^- (µM)	sd	NO_2^- (µM)	sd
Control	1	7.40	0.16	47.65	0.70	4.26	0.07
Control	28	14.80	0.49	56.26	0.03	3.91	0.02
Control	65	37.62	0.45	51.93	1.01	4.18	0.05
Cu	1	5.00	0.45	-	-	-	-
Cu	28	13.04	0.12	57.21	1.87	4.39	0.05
Cu	65	22.84	0.16	49.67	0.37	4.16	0.10
NaCl	1	5.45	0.73	46.26	0.31	3.69	0.03
NaCl	28	10.35	0.08	53.29	0.06	4.09	0.03
NaCl	65	20.88	0.08	52.88	0.49	4.03	0.05
Glucose	1	6.94	0.41	51.24	0.52	3.86	0.00
Glucose	28	7.44	0.08	69.17	4.10	3.83	0.05
Glucose	65	40.80	1.59	50.57	0.61	4.18	0.05
NH4	1	5.62	0.19	50.60	0.39	3.86	0.07
NH4	28	15.17	0.23	66.61	0.52	3.95	0.02
NH4	65	39.57	0.08	50.14	0.15	4.15	0.08

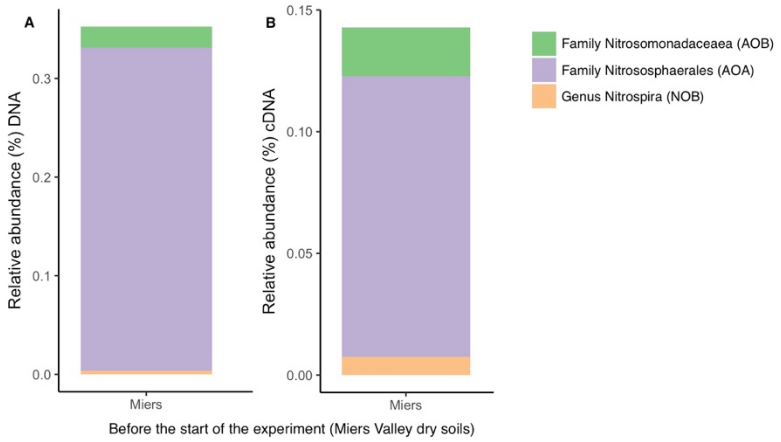

Figure S1 – Relative abundance (%) of 16S rRNA gene sequences (A) and transcripts (B) assigned to taxonomically identified nitrifying microorganisms (AOA – ammonia-oxidizing archaea; AOB – ammonia-oxidizing bacteria; NOB – nitrite-oxidizing bacteria) before the start of the manipulative experiment in Miers Valley soils.
